# Annotations

**Published:** 1898-10-01

**Authors:** 


### ANNOTATIONS.

Scarborough is to have an infectious diseases hospital, and plans have been passed at an estimated cost of £15,000.

The ninth meeting of the Italian Congress of Internal Medicine will be held at Turin on October 3rd to 7th.

The Prince of Wales's Fund has received a donation of £500 from Mr. Patrick Agnew Reid as a thanks offering.

It is stated that Ipswich is the only union in the eastern district where ophthalmia prevails in the workhouses. At St. John's Home, Ipswich, 27 cases are reported.

The introductory address at the opening session of the London School of Medicine for Women will be delivered by Dr. Walter Carr on Tuesday evening, October 4th, at 8 p.m. Mrs. Burt will distribute the prizes to the successful students.

The American papers are full of complaints of the mismanagement of the medical arrangements of the War Department during the recent war. It is affirmed that the medical necessities and food supply were totally inadequate.

The Secretary of the Local Government Board has sent a notice to Boards of Guardians stating that under the new arrangements for public vaccination, contemplated by the Vaccination Act, the use of public vaccination stations will be discontinued, except where "by reason of serious risk of outbreak of small-pox or other exceptional circumstances" it may be deemed expedient to use them.

An unfortunate case which occurred at one of the London hospitals last week shows the importance of always being awake to the possibility of what appears like hysteria being really but a symptom of some graver disorder. The patient, a domestic servant, became ill and excited, and on being taken to St. Mary's Hospital was thought to be suffering from hysteria, and treated accordingly. She afterwards, however, died, and the medical evidence at the inquest was to the effect that she was suffering from meningitis.

The typhoid epidemic still continues in Belfast. The water supply is now under examination, but, apart from the result of the analysis, the insanitary condition of many parts of Belfast is unquestionably dangerous.

Professor Ogston has resigned the post of senior surgeon to the Aberdeen Royal Infirmary, as he finds the arduous duties entailed more than he can undertake. Professor Ogston has been connected with the infirmary for thirty years, and his loss will be keenly felt.

Mr. S. R. Christophers, of University College, Liverpool, late Holt scholar under Professors Sherrington and Boyce, has been nominated by the Royal Society to serve on the Commission entrusted by the Government with the investigation of malaria in the British colonies.

On Wednesday, October 19th, at half-past seven p.m., the Bishop of London will preach at St. Paul's Cathedral to the members of the medical profession. The service, which is an annual one, is organised by the Guild of St. Luke, and will be, as in past years, a brilliant ceremony, as all doctors who are graduates or Fellows of the Royal College of Surgeons are asked to attend in academical robes. At the corresponding service last year upwards of 1,200 medical men were present, and the space under the dome of the Cathedral was largely filled with those in academical dress. The music will be rendered by a choir of over two hundred voices, provided by the London Gregorian Association, so that the whole service is likely to be most solemn, brilliant, and impressive. Admission to the space under the dome will be by ticket only.

We are asked to publish the following particulars respecting the Guild of St. Barnabas for Medical Students: Monthly services will be held in the North-west Chapel of St. Paul's Cathedral at a quarter past six p.m., on the first Wednesday in each month, commencing October 5th. Tea is served to members at a quarter to six in the Chapter House before the services. There will be a sessional corporate communion in the Jesus Chapel of St. Paul's Cathedral at half-past seven a.m., on Thursday, October 6th. The objects of the Guild are the encouragement of a high ideal of faith and practice amongst students of medicine. The Guild is open to all students who are members of the Church of England. The chaplain is Canon Newbolt, and the hon. secretary B. Bailey, Esq., 14, Woburn Square, W.C., from whom all information can be obtained.

**Muddled Consciences.**

The conscientious objector has not yet come to the end of his facetious reasons for disliking vaccination, nor have the magistrates arrived at any general conclusion in regard to the sort or amount of evidence by which they will be satisfied. Hence the dull routine of the police-courts is still occasionally relieved by the excuses of the anti-vacs. But, indeed, the efforts of the magistrates to make sure of the "conscientiousness" of the objections put forward, and to ascertain how they themselves are to be satisfied are almost equally ludicrous. A lawyer in one court went so far as to say that the magistrate must first satisfy himself that the applicant had a conscience, and then must further be satisfied that it was his conscience, whatever that was, that impelled him not to have his child vaccinated, on the ground that he believed it would be prejudicial to its health. But there is no end to the inanities which could be got out of the wording of the Act. The present state of affairs, however, will settle down before long. A phraseology will be found which will satisfy the magistrate, the shibboleth will be learned off by the objectors, perhaps will even be printed on little cards for their use, and all questions of conscience will subside.

### Insects and Disease.

Nothing could more strikingly illustrate the importance of small things than the large rôle which is now attributed to the mosquito in the etiology of some of the most serious and widespread diseases to which the human race is subject. It is truly said that what prevents the successful colonisation of many tropical countries, and what throws the greatest obstacle in the way of civilisation of and good government in vast regions of Central Africa, is not climate, not distance from home, and not unfriendliness on the part of the natives. The obstacle is malaria, and now we find that the prevalence of malaria, so far as man is concerned, depends on the mosquito, and that this pestilent little insect, in addition to irritating and annoying, is the means by which the poison of malaria is propagated and distributed. For years back botanists have known the important part played by birds in the scattering of seed, and of insects in the distribution of the pollen of plants; and it seems not unlikely that pathologists will have to recognise, in a much larger degree than has till lately been done, the large part taken by the subordinate forms of life by which we are surrounded—our cattle, our horses, our dogs and cats, our flies, our mosquitos, and perhaps even our fleas—in distributing disease from man to man, and, as is stated in regard to the mosquito and malaria, in deciding whether the extension of our empire over great areas of the globe's surface shall be possible or not.

### The Early Discharge of Lunatics.

There is and probably there will continue to be much dispute as to the meaning of the steady increase in the number of lunatics who come under the supervision of the Lunacy Commission year by year. According to the annual report of the Commissioners in Lunacy, there was an increase of 2,607 in the number under treatment between January 1st, 1897, and the same date this year. Whatever explanation may be given, the matter is a very serious one to the ratepayer. But there is an aspect of the matter which seems to us to be of an even more serious import to the community, namely, that the registered lunacy of the country is only kept within even these large figures by dint of discharging a large number after such a short detention that they soon have to be taken back again. It is stated that of the fresh admissions no less than 16·1 per cent. were cases readmitted into asylums from which they had previously been discharged, and the serious question arises whether, under the influence of pressure for space, people are not being discharged far sooner than is wise. It is admitted that a large proportion of the apparent increase in the number of lunatics is due to the growing tendency to send into asylums patients who used to be treated at home or in the workhouses, many of them old and chronic cases. Meanwhile, it seems as if, to make room for these, the more acute cases are being sent out so early that 16 per cent. of them have to be taken in again, and that the country is thus being flooded with half-cured lunatics ready to break out again at any moment. This, perhaps, may not matter very much; but unfortunately many of them return to their wives and families during their more lucid moments, and if there is any truth at all in what we are so confidently taught about heredity it is not difficult to see how lunacy is being bred amongst us.

### Occupation as an Element in Diagnosis.

A person's occupation sometimes becomes a very important factor in diagnosis when he is attacked by abnormal or ill-defined disease. This is markedly the case in regard to grooms and those whose work has to do with horses or stables, for glanders may take on curious forms and may be anything but easy of diagnosis. This was well illustrated by a case lately under the care of Dr. Sharkey, of St. Thomas's Hospital, which is reported in the *Lancet*, September 24th. There was a history of having been treated for what the patient called rheumatism—pain shooting down the right thigh to the calf—which had been looked upon as due to syphilitic (?) periostitis of right tibia, and later from a sore on the arm which had been thought to be gummatous. For these he had taken iodide of potassium and had apparently undergone some improvement, for he had ceased to attend. On admission he was found to be suffering from abdominal pain, mainly referred to the epigastrium and the adjacent part of the right hypochondriac region. In many ways the case simulated typhoid fever, but ultimately nodular swellings, on the summit of which pustules occurred, came on the face, there was an offensive discharge from the nostrils, and by cultivations the disease was shown to be glanders. In the meantime, however, the patient died. At first the enlarged spleen, the general malaise, and the moderately high temperature suggested typhoid, but the result of the serum test was negative, and later the range of temperature became very unlike that of typhoid, and that hypothesis was negatived. The course of the disease was, indeed, very anomalous throughout. The teaching of the case seems, however, to be that the fact of a man having to do with horses, even though as in this case the horse in question was believed to be quite healthy, should be taken as suggesting bacteriological search for glanders should he be attacked by any anomalous form of disease. Even external suppurating glanders is easily mistaken for broken down gummata, but internal glanders in the absence of buttons and nasal discharge may give no characteristic symptoms unless bacteriological cultures are made.

### First-Aid Dressing Packets.

The utility of first-aid packets has been very markedly shown during the recent war in Cuba. In a report made to the Surgeon-General it is stated that although the work done by the regimental surgeons and their hospital squads upon the field during battle was very effective, many dressings were applied by the officers and soldiers on the firing line, and, in some instances, by the wounded men themselves. The rarity with which suppuration has occurred is probably, to a large extent, to be accounted for by the promptness with which these dressings were applied.

### Partnership in General Practice.

If we may judge by the difficulty which seems to have been experienced on every side in finding suitable qualified men to take charge of practices during the holidays, the action of the General Medical Council in regard to the employment of unqualified assistants must be beginning to tell effectually upon medical practice throughout the country. So far as the *locum tenens* question is concerned, it has probably acted in two ways; it has dried up the supply by providing occupation for many qualified men who had before been undersold and kept out of work by their unqualified competitors, but it has also considerably increased the demand by making it impossible for those who still employ unqualified assistants in a subordinate capacity to leave them in charge, as they probably would have done previously if a chance of a holiday had cropped up during the slack season. There is no doubt that the existence of a substratum of unqualified helpers, who in times of emergency, or when holidays were wanted, could be pressed into the service, gave a great elasticity to conduct of many a practice, an elasticity which now will be lost; and one can hardly help seeing that the final effect of the action of the Medical Council must be to give a great impetus to medical partnerships, and that it will tend to turn what have been personal practices into partnership concerns. Some of the best practices in the country have for long been carried on as partnerships with satisfaction and comfort to all concerned. Such businesses remain permanently in occupation of the field; there is no trouble with assistants, for the junior does the rougher work; there is no trouble with dispensing, for the "firm" can afford a good dispenser; there is no trouble about holidays, for it can always be arranged for one partner to go away at a time; the giving of anæsthetics and the doing of small operations cease to be a worry, for all these things can be arranged for within the four corners of the "concern"; moreover, the constant introduction of fresh juniors prevents such a practice from ever getting old-fashioned, while, nevertheless, the knowledge and experience of the senior remains permanently at the disposal of his partners. Such is the ideal general practice of the future; the juniors working under the guidance and with the support of their more experienced partners, the elders gradually attaining the ease and dignity of consultants—consultants to the firm. As to the public, partnerships are greatly to their advantage, for by the extension of the system the difficulty of finding a doctor in an emergency would be greatly lessened.

### Gastric Tetany.

In view of the fact that the text-books say that the prognosis of tetany is good, it is well to be reminded, as we are by two papers in the *Lancet* for last week, that there is a form of tetany the prognosis of which is very bad indeed, namely, that which comes on in connection with dilatation of the stomach—gastric tetany. The condition is sufficiently uncommon to make it possible that many practitioners have never seen, perhaps have never heard of, such cases. Nevertheless, the association is one always to remember, especially in view of the statement which is made that 75 per cent. of such cases die; for one might easily be tempted to make light of the early symptoms, and thus be led to give an erroneous prognosis. In ordinary cases, tetany begins rather suddenly, the first symptoms, at any rate in adults, generally being numbness and tingling in the fingers. This is soon followed by tonic contraction of the fingers and thumbs, the fingers being extended at the phalangeal joints and slightly flexed at the metacarpo-phalangeal joints, while the thumbs are bent into the palm. The wrists and elbows become slightly flexed, and the lower limbs are also sometimes affected, the toes being adducted and flexed, and the position of the foot being like that assumed in talipes equino-varus. Occasionally the contraction spreads to other muscles involving those of the head and neck, and even the diaphragm. The spasm lasts a varying time; sometimes it continues for several hours and then passes off, to return again and again, perhaps for several months. Gastric tetany, however, as described in the papers by Dr. Trevelyan and Dr. Souttar McKendrick, is a much more acute affair, lasting only a few hours or days, and ending in death; and although in some of the cases which Dr. Trevelyan has so carefully collated from many sources the disease was not so severe, it does in its general features stand very far apart from the milder forms of the disease, which are more commonly met with. The origin of this gastric tetanus has been put down to various causes. It has been thought to be reflex, but, as Dr. Trevelyan says, the application of the reflex theory to the causation of disease has of late been narrowed down to within very modest limits, and must not be too readily accepted. Then it has been thought to be due to the draining of the water from the tissues in consequence of its being poured so largely into the dilated stomach, but there are cases in which this explanation cannot hold good. Of the several theories which have been put forward, none seem so probable in the light of modern knowledge as that which attributes the production of gastric tetany to an auto-intoxication—an intoxication arising from substances retained in the dilated stomach. If we are to accept this intoxication theory, as we feel almost bound to do, the therapeutic indication is at once and repeatedly to wash out the stomach on the occurrence of the slightest sign of tetany, so as to remove every trace of its decomposing contents.

### Pauperism in Whitechapel.

All honour to the Board of Guardians for Whitechapel, for they have faced and dealt with the problem which presents itself to every board where those who support the pauper are themselves to be reckoned among the poor! During the last twenty-eight years they have resolutely set their faces against the giving of those doles in the way of outdoor relief which the experience of all students of the problems of poverty shows to be a mere encouragement of mendicity, without any permanently good effect on the condition of those who receive them. In the sixth week of the quarter ending Lady Day, 1871, there were 2,568 outdoor paupers receiving aid from the Whitechapel Guardians, while the number of indoor paupers in the workhouse was 1,410. They then decided to restrict as far as possible the giving of outdoor relief. The result is that at the same week of 1898 there are only 24 persons in receipt of outdoor relief, of whom 20 are boarded-out children. This has been done without adding to the number of indoor paupers, who at the same date numbered 1,413, that is, only three more than in 1871, and even this increase would not be found, in the opinion of the clerk of the board, but for "the increase of the sick poor under the separate infirmary system." In effect, there seems to be no doubt that the Whitechapel Guardians have largely decreased the amount of pauperism under their control. This was certainly their duty to their constituents, who are for the most part little able to support others beside themselves. The next question is, Have they saved the purses of their constituents at the expense of any who needed aid? It is doubtful if they have. In the first place, the weekly half-crown very rarely does any good to the recipient. If it is used to pay the rent, it may enable the pauper to accept work at wages which are not remunerative to those who are not subsidised. The effect of this on wages generally every economist can tell. Or the money is spent in food, which, being consumed, leaves the consumer no farther off from pauperism than before. These doles do no lasting good; and as it is not the province of the poor law to do more than relieve present destitution, it is not to be expected that they should. But there are in London, as all over the country, many organisations which are intended to take up and deal with individual cases as poor law officers cannot. If the guardians are in touch with these institutions, whether those in connection with particular churches, or those based on purely secular philanthropy, they can often put a deserving poor person in the way of receiving help, not merely of a more substantial and permanent nature than they are empowered to give, but such as may help the recipient to rise above the status of pauperism. Such help, because given from more personal reasons, with a more human touch, is likely to be more appreciated than poor law relief; while, at the same time, the consciousness that it is given not as a right, but as a favour, will tend to keep the recipients from being guilty of any conduct that may serve to forfeit it. Speaking generally, the deserving poor may be safely left to private kindness, guided, perhaps, by the unsentimental eye of poor law officials, while the undeserving poor should not be trusted with doles of money which they can spend as they choose. It seems that at present there is a reaction threatened against the sound principles which have done so much to eliminate the outdoor pauper from Whitechapel. This is to be regretted, and we fear that if this reaction has its way, the ratepayers will be poorer, while the deserving poor of the district will be no richer.

### Aseptic Midwifery.

The question of aseptic midwifery is one which excites an interest which is not lessened by the fact that no general consensus seems yet to have been arrived at as to what is meant by the term. The extremists no doubt do good by putting their case fully and logically before their professional brethren. No sooner, however, do they do so than "common sense" rushes in with a long list of successful cases secured by means of "ordinary cleanliness." Dr. Jardine, physician to the Glasgow Maternity Hospital, in describing at Edinburgh the proceedings necessary in what he spoke of as "aseptic midwifery," said: "When the hands have been thoroughly sterilised, you must see to your patient, and get the field of operation made as aseptic as possible. The external genitals, with their many folds and abundance of hairs, make as fine a lair for the lurking microbe as one could well imagine. The parts must be thoroughly cleansed with soap and water and an antiseptic. Lysol I find the best of all for this. I am perfectly well aware that it is impossible in this way to render the parts absolutely aseptic, but that is no reason why we should not attempt to cleanse them as much as possible. Do this yourself. If you were going to do an abdominal section, you would never dream of making your incision before rendering the skin as aseptic as possible, neither would you trust to anybody else doing this for you. No examination should be made until the parts are clean; sterilise your hands, and do not allow the examining hand to touch anything before the fingers are inserted into the vagina. The woman's buttocks and external genitals must be in full view to enable you to do it properly. No pure-minded woman will object to this. To the pure all things are pure." This then represents the preliminary stage of an aseptic confinement, and as the labour may last many hours, and an internal examination—if more than one is to be made—may take place at long intervals, during which the patient may have been up and about, we may presume that this process is to be repeated more than once during the course of an accouchement. Now we quite recognise the desirability of these proceedings, and we quite see that in any long series of cases—in which here and there an unsuspected septic vagina is sure to be met with—the systematic carrying out of such a method in every case will lower the mortality. Nevertheless we are not altogether surprised that this teaching has drawn forth a protest, or that this insistence that the doctor should do himself what has been so commonly relegated to the nurse should have been made an occasion for bringing the details themselves into question. Dr. Allen McCulloch has had in his general practice 1,753 cases without a maternal death, and, so far as he can now remember, without a single case of septic inflammation, and he raises the standard of revolt against such elaboration as Dr. Jardine advocates and against the "hyperæstheticism" which is showing itself in this branch of medicine. He says, "What self-respecting woman, outside an institution, would submit to have the genitals exposed and washed and scrubbed by the doctor? How many practical obstetricians in this country could say they ever did such a thing or thought it was necessary as a part of their midwifery practice?" And he goes on to say that his results are as good as those of Dr. Jardine; indeed, he challenges him to say if they are not better. We think that a middle course is possible, and that this middle course lies in the better education of the monthly nurse. In the meantime we cannot but see that the position taken up by Dr. McCulloch tends to bring other and wider issues into the field, and that the form of his protest will bring up the question of the midwife over again. If the extreme teaching in regard to aseptic midwifery is not to be accepted because the precautions insisted on are unnecessary, well and good. The two parties must appeal to experience and abide the result. But if aseptic midwifery is to be declined because it is not seemly for the medical man to carry out its details, another question comes to the front directly, namely, Who ought to do the midwifery?

